# How I do it – exoscopic retractorless anterior skull base reconstruction using a pedicled pericranial flap for post-traumatic CSF leak

**DOI:** 10.1007/s00701-026-06961-w

**Published:** 2026-06-17

**Authors:** Michael Veldeman, Mark Johannes Marinus Houmes, Hans Clusmann, Hussam Hamou

**Affiliations:** Department of Neurosurgery, RWTH Aachen University Hospital, Pauwelsstrasse 30, 52074 Aachen, Germany

**Keywords:** Anterior cranial fossa, CSF fistula, Rhinoliquorrhea, Bifrontal craniotomy, Pericranial flap, Skull base repair, Traumatic brain injury

## Abstract

**Background:**

Traumatic anterior cranial fossa fractures complicated by cerebrospinal fluid (CSF) fistulas carry the risk of ascending meningitis.

**Method:**

We describe our standardised technique for anterior cranial fossa repair via a bifrontal craniotomy, including preparation of a pericranial flap, closure of the frontal sinus, bilateral olfactory nerve dissection, and intradural pericranial reconstruction.

**Conclusion:**

A systematic, anatomically informed approach enables durable watertight repair with acceptable morbidity. If surgery is properly timed after cerebral swelling has resolved, and CSF is drained, retractorless surgery prevents injury to the inferior face of the frontal lobes.

**Supplementary Information:**

The online version contains supplementary material available at 10.1007/s00701-026-06961-w.

## Relevant surgical anatomy

Traumatic fractures of the anterior cranial fossa floor typically occur along its thinnest and most vulnerable components: the orbital plates of the frontal bone, and cribriform plate of the ethmoid [1, 2]. These structures are intimately related to the frontal sinus anteriorly and the ethmoid air cells centrally, such that fractures which disrupt the dura may establish a direct communication between the non-sterile paranasal sinuses and the subarachnoid space.

The olfactory nerve fascicles traverse the cribriform plate’s foraminae. The olfactory bulbs lie on the cribiform plate and continue posteriorly as olfactory nerves which are adherent to the inferior surface of the frontal lobes. Traumatic olfactory injury most commonly occurs at the level of the cribriform plate, where the delicate olfactory fila are disrupted by the fracture. However, a more proximal avulsion of the olfactory nerve from the undersurface of the frontal lobe is also possible, particularly in the setting of severe deceleration forces, and may be encountered intraoperatively during subfrontal dissection. Subarachnoid olfactory dissection, necessary to access and reconstruct the anterior fossa floor, carries an inherent risk of olfactory impairment.

The caecum of the superior sagittal sinus originates at the crista galli. Although narrow, it must be ligated and divided during the bifrontal approach to allow elevation of the dura and adequate exposure of the anterior fossa floor.

Along the superior orbital rim, the supraorbital nerve and artery exit through the supraorbital foramen or notch.

## Description of the technique

Thin-cut bone-window computed tomography (CT) images in all three planes are reviewed to delineate the course of the fracture, and the presumed site of dural tearing. In patients with frontal contusional hemorrhages, subfrontal surgery should be deferred until swelling has subsided. Premature surgery increases the risk of retraction injury. In the interim, rhinoliquorrhoea can be managed with lumbar CSF drainage and prophylactic antibiotics.

**Fig. 1:**
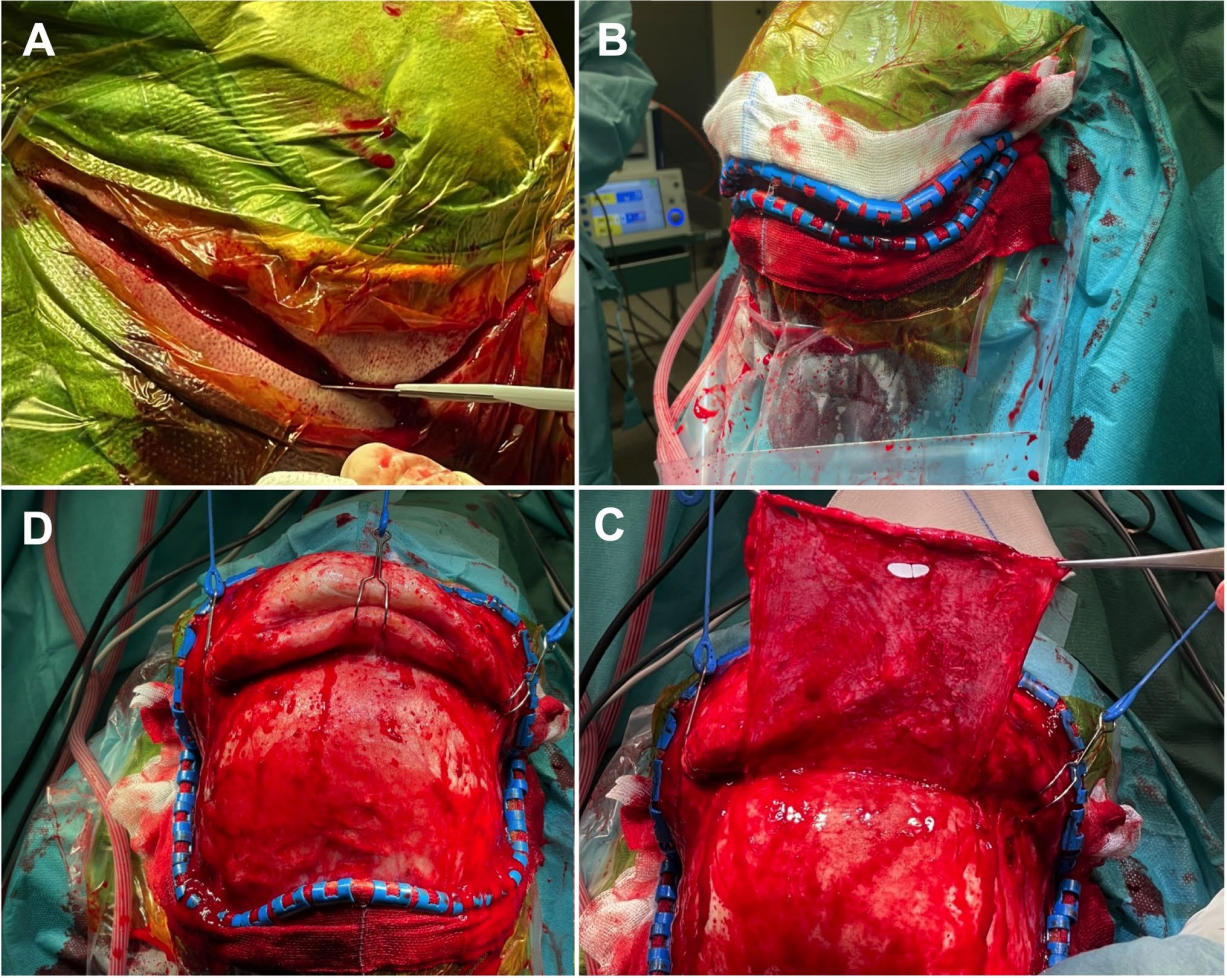
Macroscopic steps of the surgical procedure. **A &B**. Bicoronal skin incision. Dissection is performed in the plane between the subcutaneous tissue and the galea aponeurotica in order to preserve the vascularized pericranium for subsequent reconstruction **C**. Intraoperative view after reflection of the scalp flap showing the bicoronal incision and the surgical field prepared for elevation of the pericranial flap. The circular opening in the flap as a result of injury during dissection is closed with 4–0 Vicryl sutures prior to intracranial deployment. **D**. Operative field after elevation of the pericranial flap, demonstrating exposure of the frontal bone prior to bifrontal craniotomy and anterior skull base reconstruction

The patient is positioned supine. Mild retroflexion facilitates bilateral frontal lobe elevation. A standard ear-to-ear bicoronal incision is made. The incision is kept curvilinear rather than straight to reduce skin tension when the flap is reflected anteriorly (Fig. 1A & B).

During the skin incision, care is taken to incise only skin and subcutis, sparing the epicranial aponeurosis. Skin hooks are used to retract the skin flap. A No. 21 scalpel blade is used for sharp dissection between the dense subcutaneous connective tissue and the epicranial aponeurosis. Tension on the skin hooks is incrementally increased as the dissection progresses anteriorly toward the superior orbital rim.

Care is taken to protect the neurovascular contents of the supraorbital foramen and supraorbital notch, including the supraorbital nerve and artery, which provide sensory innervation and perfusion to the anterior scalp respectively (Fig. 1C).

The epicranial aponeurosis, loose areolar tissue, and pericranium are sharply incised along the posterior margin of the skin incision. Two parallel linear incisions are then made, slightly above the superior temporal lines. Using a periosteal elevator, the pericranial flap is dissected off the skull (Fig. 1D).

## Craniotomy and closure of the frontal sinus

The temporalis muscle is incised and a small flap is reflected to expose the pterion. Burr holes are placed at both MacCarty keyholes. A third burr hole is placed on the midline frontal bone, approximately 4–5 cm above the glabella. The dura is carefully dissected and elevated from the inner table.

Craniotome cuts are made completing the bifrontal bone flap. After removal, the bone flap is carefully inspected for involvement of the frontal sinus. If mucosa is encountered, it is stripped. The posterior wall and floor of the anterior cranial fossa are then drilled back an additional 3–5 mm using a diamond burr and/or bone rongeurs. The operative field is irrigated and disinfected.

The pericranial flap is now sutured to the frontobasal dura. A running 4–0 Vicryl suture is placed to create a watertight barrier between the intracranial compartment and the frontal sinus. Once the frontal sinus is definitively closed, the surgeon changes gloves, and only unused instruments are used for all subsequent intradural work. This staged approach minimizes the risk of intracranial contamination from paranasal sinuses.

## Dural opening and olfactory nerve dissection

**Fig. 2:**
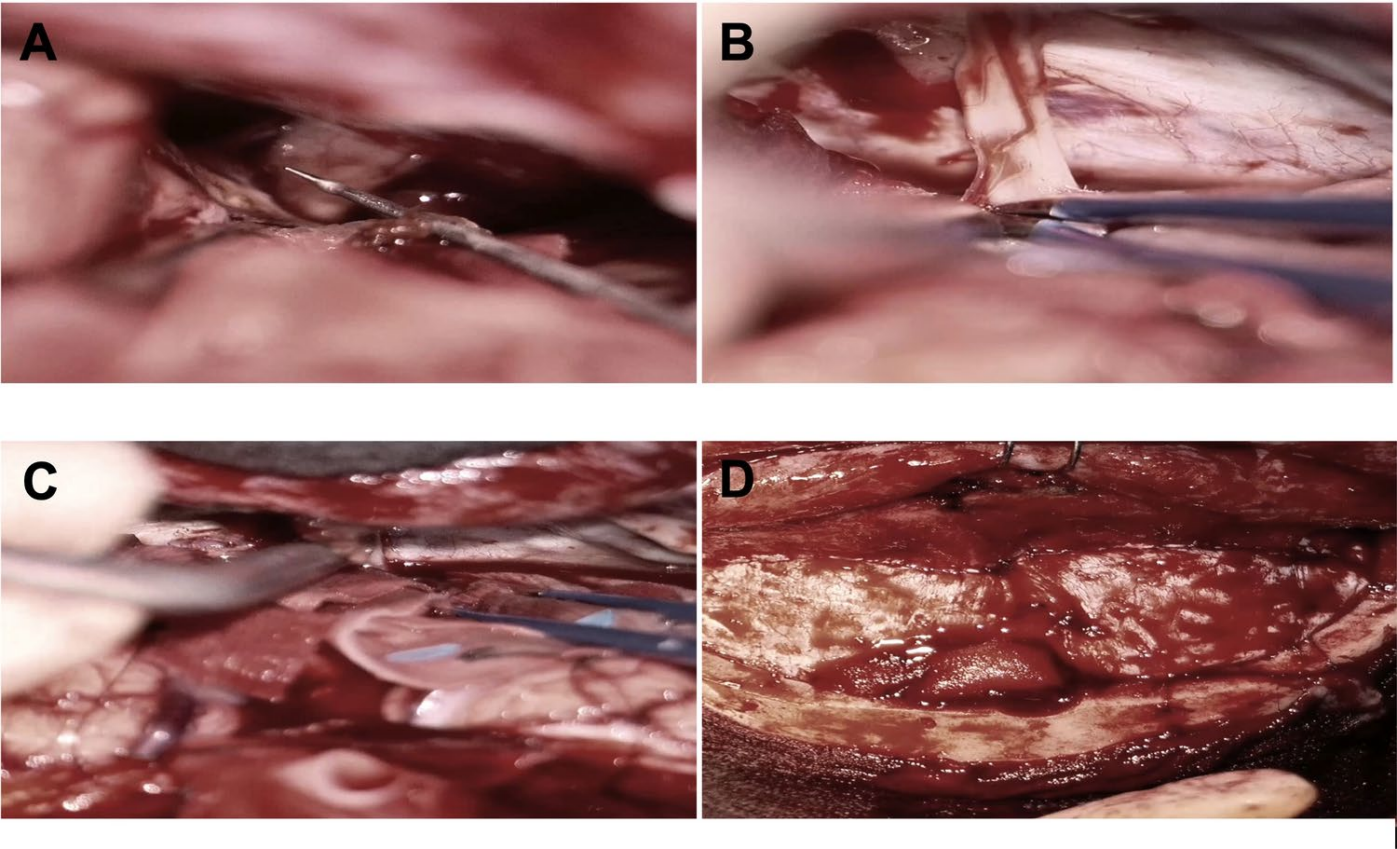
Microscopic steps of the anterior skull base reconstruction. **A**. Opening of the opticocarotid cistern to allow cerebrospinal fluid drainage and achieve brain relaxation during the subfrontal approach. **B**. Careful microsurgical dissection of the right olfactory nerve from the undersurface of the frontal lobe. **C**. Intraoperative overview of the anterior skull base after bilateral subfrontal exposure. The right olfactory nerve remains intact following dissection (below the suctioning device). The left olfactory nerve had been avulsed as a result of the initial trauma, and only the proximal stump posterior to the olfactory bulb was encountered intraoperatively (see Supplemental Video). **D**. Final reconstruction of the anterior skull base. The pedicled pericranial flap is sutured to the superior edge of the dura to achieve a watertight closure prior to repositioning of the bifrontal bone flap

Under magnification, a linear dural incision is made 3–5 mm above the previously sutured pericranial flap. In the provided Supplementary Video, we make use of an exoscope for intradural dissection. Exoscopic magnification carries the advantage of a higher depth of field, cold illumination, and improved surgeon ergonomics. The caecum of the superior sagittal sinus is ligated with two 3–0 nylon sutures, coagulated, and divided. The falx cerebri is coagulated and incised. On the side with the least anticipated scarring or swelling, a unilateral subfrontal approach is performed first. The opticocarotid cistern is identified and opened to drain cerebrospinal fluid, achieving brain relaxation and reducing the need for retraction (Fig. 2A).

The ipsilateral olfactory nerve is then identified along the inferior surface of the frontal lobe (Fig. 2B&C). Using a combination of blunt and sharp dissection, the arachnoidal attachments of the olfactory nerve are dissected and the nerve is freed from the undersurface of the frontal lobe distal-to-proximal. This procedure is then repeated on the contralateral side.In the case presented in the Supplementary Video, the left olfactory nerve had been avulsed proximally from the undersurface of the frontal lobe as a result of the initial trauma; only its proximal stump was encountered upon subfrontal elevation, consistent with a pre-existing injury rather than an iatrogenic one (see also Fig. 2C).

## Pericranial fixation and closure

The pericranial flap is now folded intracranially and laid carefully under both frontal lobes to cover the anterior fossa floor. Adequate reach of the flap must be visually confirmed. Holding suture may be place at the level of the sphenoid wing or alternatively fibrin glue can help immobilized the flap.

The dural incision is closed by suturing its edge to the pericranial flap, creating a watertight dural reconstruction (Fig. 2D). The bone flap is repositioned and fixed with titanium dog-bone plates and screws. Craniotomy edges and the frontal burr hole are filled with methylmethacrylate cranioplasty cement to optimise the cosmetic result.

## Indications

- Persistent rhinoliquorrhoea despite an adequate trial of conservative management (typically 7–10 days of lumbar CSF drainage and bed rest)
- Meningitis attributable to an anterior fossa CSF fistula
- Significant skull base defects/dislocated fractures, identified on imaging with high likelihood of dural interruption
- Patient preference for definitive surgical repair following informed counselling regarding the risks of conservative versus operative management

## Endonasal versus endocranial approach

The choice between an endonasal endoscopic and an open endocranial approach is guided primarily by the size, location, and complexity of the defect. Endonasal endoscopic repair is preferred for small, well-localised defects involving the sphenoid sinus or an isolated posterior table of the frontal sinus, where direct visualisation and watertight closure can be achieved without craniotomy. Once the ethmoid sinuses are involved, or when fractures are displaced or span multiple compartments, endonasal closure becomes technically more demanding and the risk of incomplete repair increases. In such cases—and particularly when bifrontal or pan-sinus involvement is present, or when there is significant intracranial pathology requiring concurrent treatment—an open endocranial approach via bifrontal craniotomy, as described in this article, is preferred.

## Postoperative management

A lumbar drain is not routinely placed following repair of linear fractures with small dural defects. For larger reconstructions, a lumbar drain may be used for 3–7 days postoperatively to reduce CSF pressure across the repair during the critical healing period. In an uneventful postoperative course, patients are typically discharged after 3–5 days. Wound staples or sutures are removed at one week. Routine postoperative cranial imaging is not performed in the absence of new neurological deficits or clinical concern.

## Limitations

- Bilateral olfactory nerve dissection carries the risk of anosmia or hyposmia, which may be permanent.
- Despite staged instrument and glove changes, the proximity of the paranasal sinuses to the operative field carries a residual risk of intracranial infection.
- Devascularisation of the pericranial flap, most commonly due to prior trauma, previous incisions, or inadvertent perforation during harvest, may compromise durable watertight repair.
- In the acute setting, bifrontal contusional edema may preclude safe subfrontal access. Surgery should be deferred in such cases.
- Complex fractures involving the posterior wall of the frontal sinus or the sphenoid sinus may require additional or alternative reconstructive strategies beyond those described here.

## How to avoid complications

- Defer surgery until cerebral swelling has resolved; use lumbar drainage and serial imaging to guide indication and timing.
- In retractorless surgery, the suction device and bipolar forceps function as dynamic retractors reducing the risk of iatrogenic contusions.
- Protect the supraorbital neurovascular bundle throughout the subfrontal dissection.
- Open the opticocarotid cistern early to obtain brain relaxation and minimise frontal lobe retraction.
- Minimise traction on the olfactory nerves; sharp arachnoidal dissection under high magnification reduces traction injury.
- Perform strict staged contamination control: change gloves, and use fresh instruments before entering the intradural compartment.

## Specific information for the patient

Patients undergoing anterior cranial fossa repair for traumatic CSF fistula should be counselled on the following points:

- The primary goal of surgery is to provide a durable watertight seal between the intracranial compartment and the paranasal sinuses, thereby eliminating the risk of ascending bacterial meningitis.
- A bifrontal skin incision (ear to ear, hidden within the hairline) is required; the resulting scar is generally well concealed once hair has regrown.
- Loss or reduction of smell (anosmia or hyposmia) is a recognised and relatively common consequence of this operation, resulting from the necessary surgical dissection of the olfactory nerves. This may be permanent.
- Temporary numbness or altered sensation in the forehead (supraorbital nerve territory) may occur and usually resolves over weeks to months.

## Key points summary

1. Traumatic anterior cranial fossa fractures with CSF fistula carry a risk of ascending meningitis and often require surgical correction when conservative management fails.
2. Thin-cut multiplanar CT imaging is mandatory for preoperative planning; surgery should be deferred in the presence of cerebral contusional swelling.
3. If in doubt whether olfaction is (partially) injured by the fracture/trauma, a smell test can help to guide patient counselling.
4. Staged contamination control before intradural work reduces the risk of intracranial infection.
5. Early opening of the opticocarotid cistern provides brain relaxation, enabling a safe and largely retractor-free subfrontal approach.
6. Retractorless surgery only using the suctioning device and bipolar forceps reduces the risk of iatrogenic contusions.
7. Meticulous bilateral olfactory nerve dissection under high magnification, using sharp techniques, minimises traction injury; nevertheless, permanent anosmia remains a risk.
8. Watertight dural closure is achieved by suturing the dural edge to the intradurally positioned pericranial flap; fibrin glue and gelatin sponge provide additional reinforcement.
9. Craniotomy edges and the frontal burr hole are filled with methylmethacrylate cranioplasty cement for a better cosmetic result.
10. Depending on the size of the dural defect, a temporary postoperative lumbar drain can be considered.

## Supplementary Information

Below is the link to the electronic supplementary material.

Supplementary file1 (MP4 57989 KB)

## Data Availability

No datasets were generated or analysed during the current study.

## Abbreviations

CSF: Cerebrospinal fluid
CT: Computed tomography
